# Supplementary figures and images for: Lycopene inhibits ER stress and apoptosis while modulating PI3K/AKT and enhancing antioxidant and anti-apoptotic proteins
Source: PLoS One. 2025 Dec 23;20(12):e0339565. doi: 10.1371/journal.pone.0339565 (PMC12725552; doi:10.1371/journal.pone.0339565)

Fig3 A

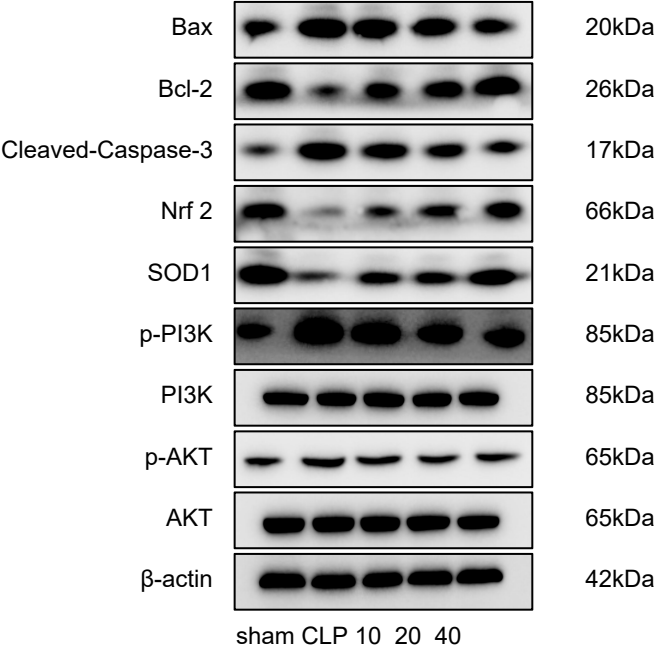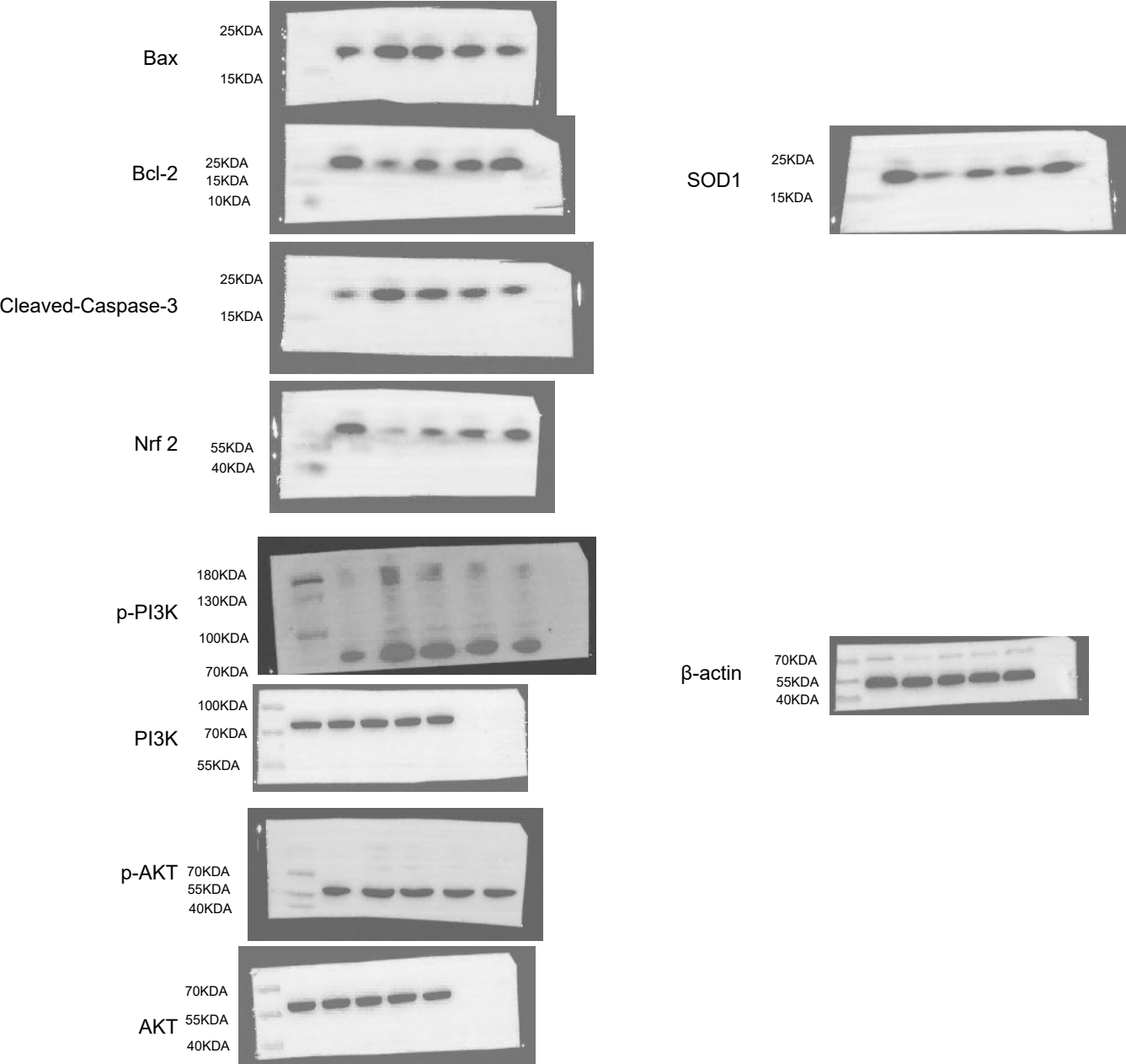

Fig7 A

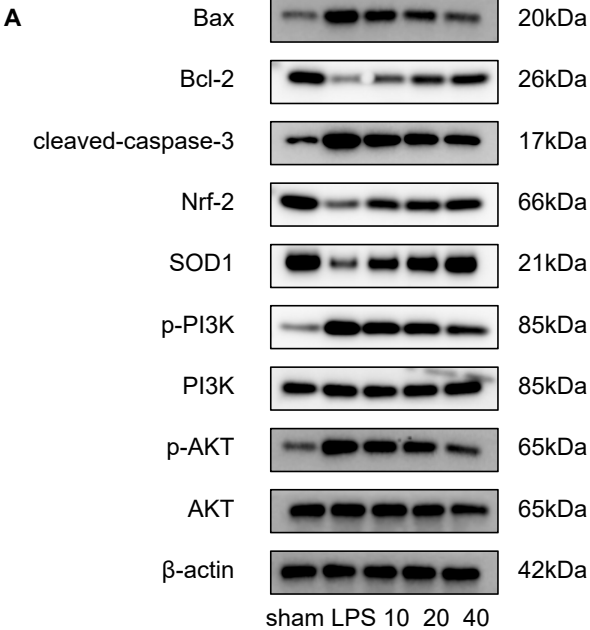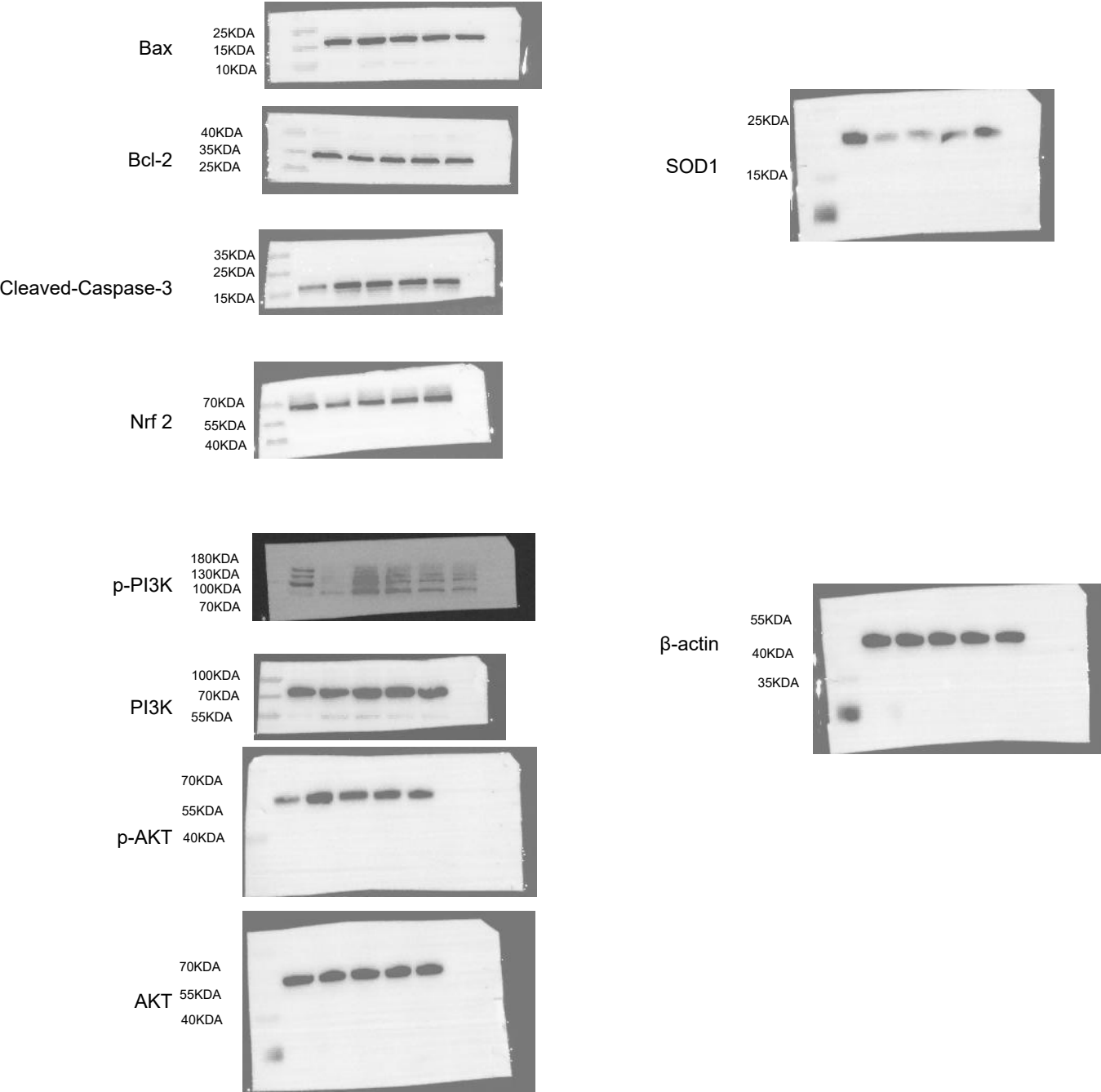

Fig7 B

B

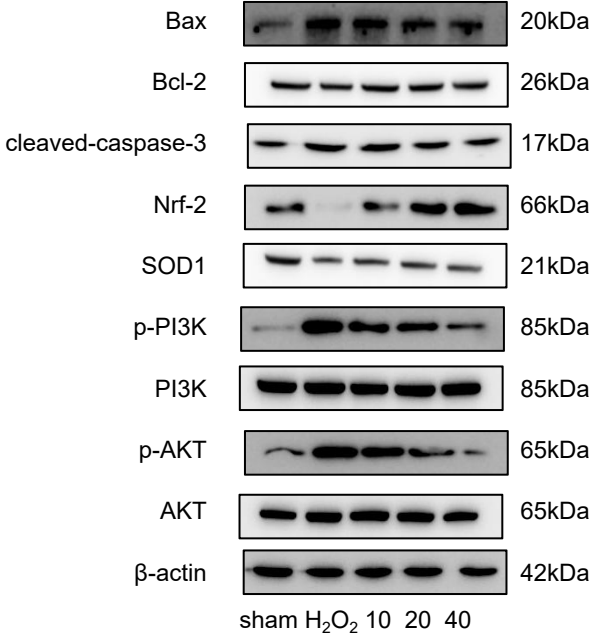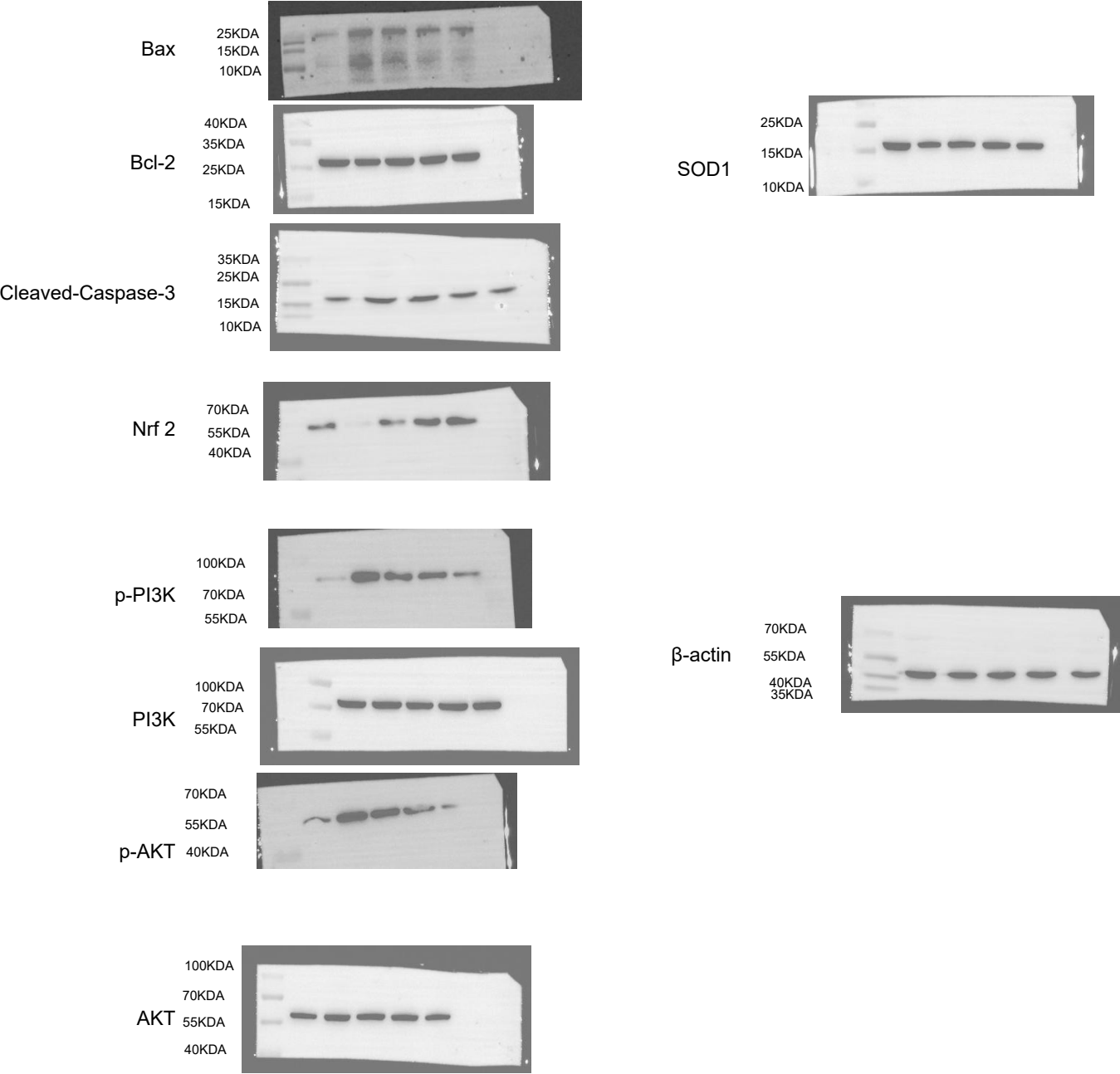

Fig8 A

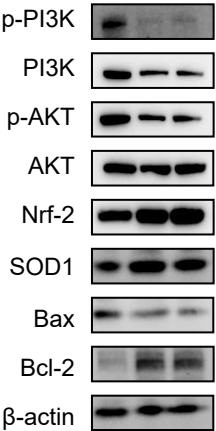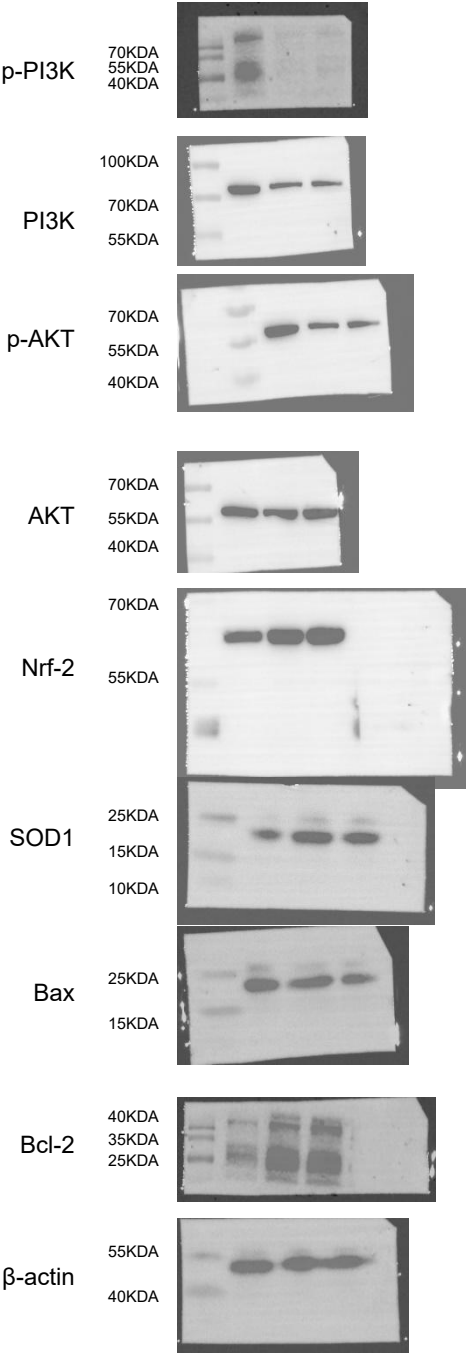

Fig8 B

B

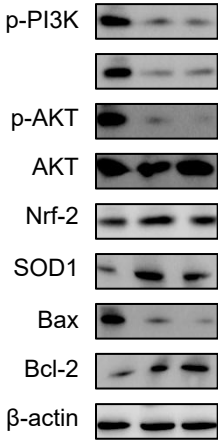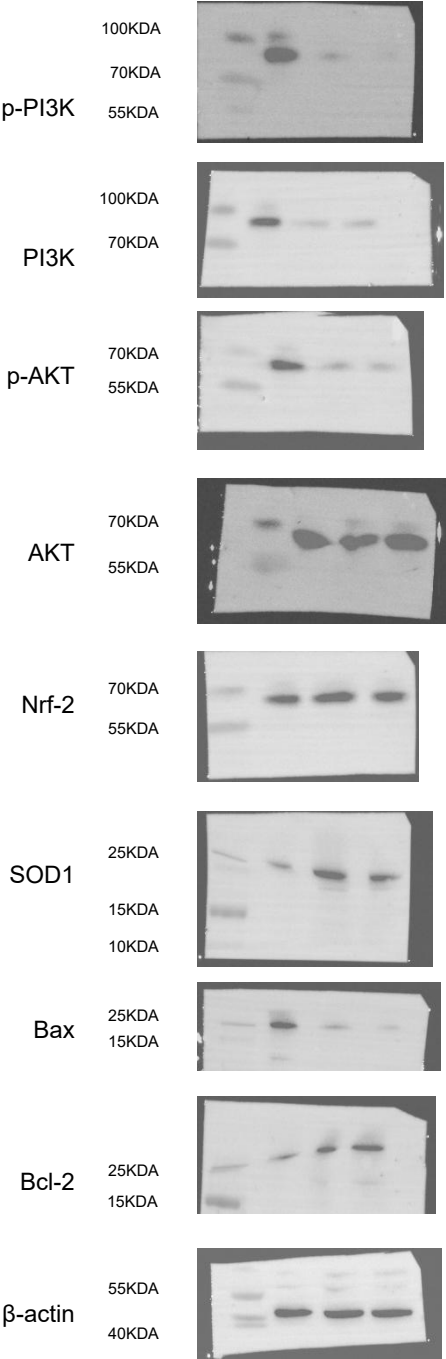

Fig8 C

c

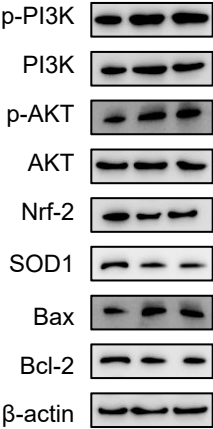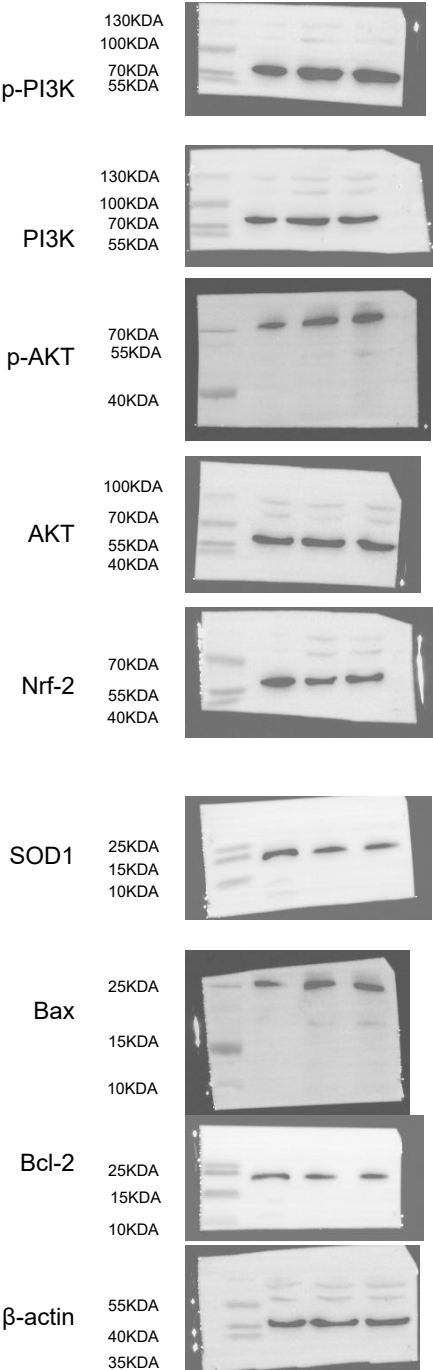

Fig8 D

D

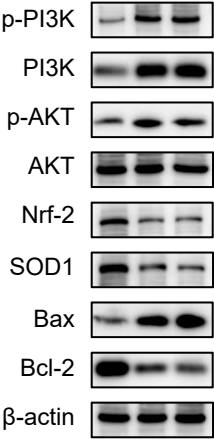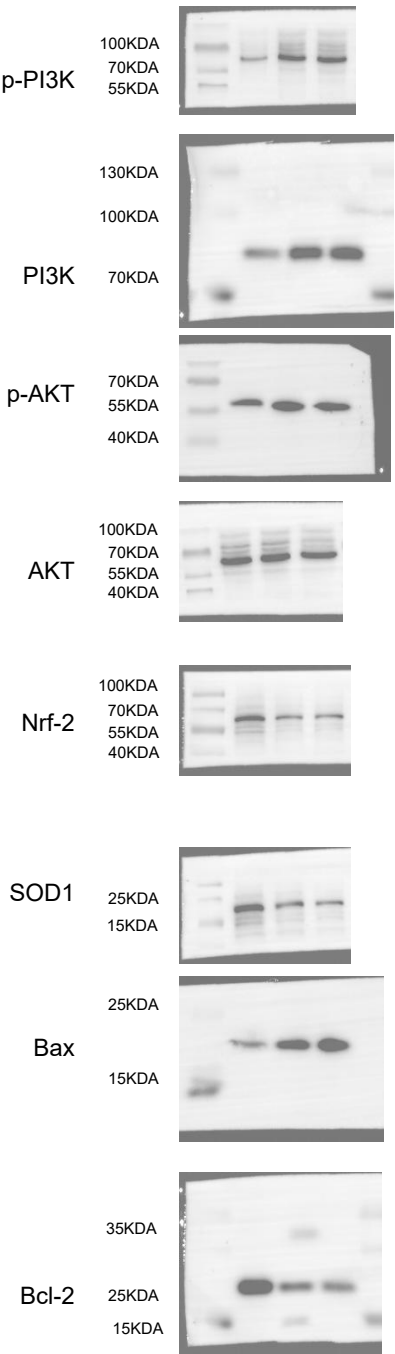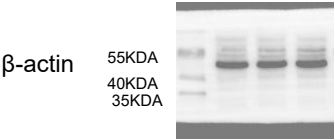

Supplement: S1 File — (PDF) [file pone.0339565.s001.pdf]
